# The novel immune-related genes predict the prognosis of patients with hepatocellular carcinoma

**DOI:** 10.1038/s41598-021-89747-7

**Published:** 2021-05-21

**Authors:** Lunxu Li, Shilin Xia, Xueying Shi, Xu Chen, Dong Shang

**Affiliations:** 1 Department of General Surgery, The First Affiliated Hospital, Dalian Medical University, Dalian, 116011 Liaoning China; 2 Clinical Laboratory of Integrative Medicine, The First Affiliated Hospital, Dalian Medical University, Dalian, 116011 Liaoning China

**Keywords:** Cancer models, Tumour biomarkers, Tumour immunology

## Abstract

Hepatocellular carcinoma (HCC) is one of the main causes of cancer deaths globally. Immunotherapy is becoming increasingly important in the cure of advanced HCC. Thus it is essential to identify biomarkers for treatment response and prognosis prediction. We searched publicly available databases and retrieved 465 samples of genes from The Cancer Genome Atlas (TCGA) database and 115 tumor samples from Gene Expression Omnibus (GEO). Meanwhile, we used the ImmPort database to determine the immune-related genes as well. Weighted gene correlation network analysis, Cox regression analysis and least absolute shrinkage and selection operator (LASSO) analysis were used to identify the key immune related genes (IRGs) which are closely related to prognosis. Gene set enrichment analysis (GSEA) was implemented to explore the difference of Kyoto Encyclopedia of Genes and Genomes (KEGG) pathway between Immune high- and low-risk score groups. Finally, we made a prognostic nomogram including Immune-Risk score and other clinicopathologic factors. A total of 318 genes from prognosis related modules were identified through weighted gene co-expression network analysis (WGCNA). 46 genes were strongly linked to prognosis after univariate Cox analysis. We constructed a seven genes prognostic signature which showed powerful prediction ability in both training cohort and testing cohort. 16 significant KEGG pathways were identified between high- and low- risk score groups using GSEA analysis. This study identified and verified seven immune-related prognostic biomarkers for the patients with HCC, which have potential value for immune modulatory and therapeutic targets.

## Introduction

Hepatocellular carcinoma (HCC) is known as the third cause of cancer death worldwide and its incidence continues to rise^1^. HCC can be induced by various risk factors, such as hepatitis B/C virus infection, nonalcoholic steatohepatitis (NASH), alcoholism, and smoking^2^. The patients with HCC often accompanying cirrhosis and many molecular pathways deregulation^3^. Traditional treatment methods for HCC patients have shown poor clinical efficacy for a long time^4^. Curative approaches for HCC including: surgical resection、orthotopic liver transplantation and locoregional therapies. While most HCC patients were already at advanced status when received the diagnoses and were not amenable to curative resection or ablation. Thus palliative treatments such as: trans-arterial approaches and systemic therapies are particularly important for such patients^5^. Sorafenib has been the only first-line therapy for advanced HCC patients for more than 10 years, while the reduction in the risk of death was limited as the median time to progression was 5.5 months and median overall survival was 10.7 months^6–8^. Recently immunotherapy is becoming the new standard treatment for advanced HCC patients all around the world^9^. Some clinical studies have shown that Nivolumab therapy can provide demonstrable responses for a subset of advanced HCC patients^10^. According to the 2020 American Society of Clinical Oncology guideline, Atezolizumab + bevacizumab has been recommended as the new first-line treatment for most advanced HCC patients^11^. This management has shown superior efficacy including higher objective response rates and median survival compared with sorafenib based on a global, open-label, phase 3 trial^12^. Combination of immune checkpoint inhibitors and kinase inhibitors will soon become a cornerstone^13^. While some early-phase clinical trials indicated that the combination therapy may increase the toxicity of individual agents^14,15^. In addition, the response rate of immunotherapy at the present stage is limited, with the objective response rate generally failing to exceed 20%. Exploring strategies to maximize patient response 、striving to better predict and choose patients who are likely to respond are the development directions of HCC immunotherapy. Therefore, novel biomarkers for prediction of treatment response are critical to develop and optimize new management strategies^16^.

Gene microarrays and RNA-sequencing technology combined with bioinformatics analysis have identified several prognostic biomarkers for cancer diseases these years^17–21^. Some immune-related prognostic signatures showed strong prediction ability. For example, Huang et al.^22^ constructed an immune related gene (IRG) prognostic classifier consisting of PSME1, CDC42, CMTM6, CXCR6, CD8B, HLA-DQB1, HLA-C, and TNFSF13 based on GEO data for melanoma patients. Similar IRGs prognostic signatures have been reported for colorectal cancer^23^, lung adenocarcinoma^24^ and gastric cancer^25^, as well.

**Figure 1:**
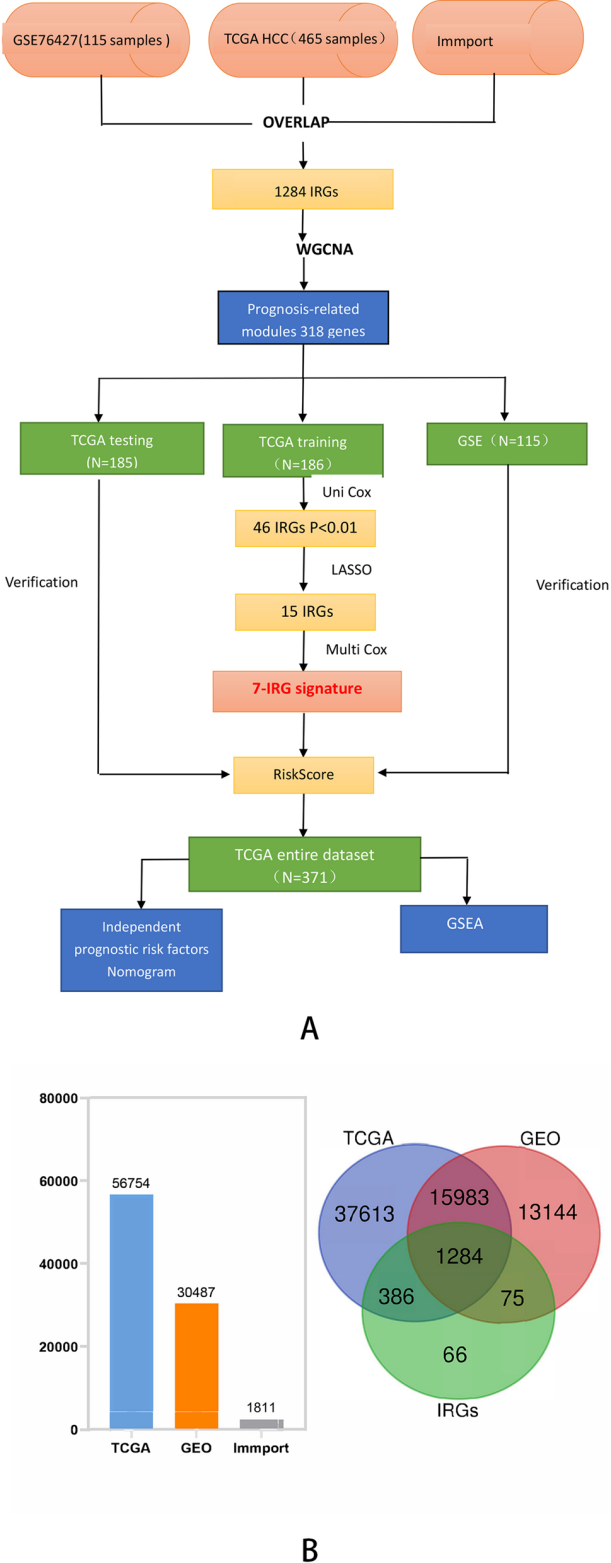
**(A)** Flow chart of the present study. **(B)** The common IRGs were visualized by Venn diagram and Histogram.

In this study, we analyzed the HCC gene expression profiles in The Cancer Genome Atlas (TCGA, http://www.cancer.gov/tcga) database using weighted gene co-expression network analysis (WGCNA). Genes from the significant prognostic-related modules were further computed for Cox regression and Lasso regression and constructed an IRG prognostic signature consisting of seven genes (NR1D1, HDGF, LMBR1, PRDX1, NR6A1, EPO and DCK). In addition, we analyzed the IRG signature by intergrating other clinical information like tumor stage, grade, patient’s age, gender and the IRG signature was confirmed as an independent prognostic indicator for HCC. The flow chart of this study was shown in (Fig. 1A). Our study established an immune-related signature for HCC patients and provided information of subsequent personalized diagnoses and treatment strategies of HCC.

## Materials and methods

### Data collection and immune related genes selection

The GSE76427 gene expression profiles were retrieved from the Gene Expression Omnibus (GEO: https://www.ncbi.nlm.nih.gov/geo/) database and the samples without follow-up information were excluded. The GEO expression file was normalized for further analysis. The HCC RNA-seq data was retrieved from TCGA database including 407 HCC samples and 58 normal samples. The TCGA dataset was normalized using the FPKM method^26^. To eliminate the batch effect of GEO and TCGA dataset, R package “sva” was applied. A total of 1811 IRGs (Table s1) related to human cancers were identified through the Immunology Databases and Analysis Portal (ImmPort) database (https://www.immport.org/home^27^. The common immune-related genes among the three above datasets were chosen.

### Weighted gene co-expression network analysis

Weighted gene co-expression network analysis (WGCNA) was performed in TCGA dataset, and the power exponential weighting of gene correlation coefficients was used to reveal the relationships among different genes^28^. We also explored the relevance among the clinical phenotype with gene modules. Module Membership (MM) represented the Pearson correlation coefficient of the module’s first principal component and module genes expression. Gene significance (GS) represented the level of linear correlation between clinical phenotype and module genes expression.

### Construction and verification of the IRG signature

The HCC patients’ clinical information was retrieved from TCGA database and the samples without overall survival (OS) and survival state information were excluded. A total of 371 samples were brought into survival analysis. Aiming to make the established prognostic signature have better generalization, the samples were divided into training dataset (186 samples) and test dataset (185 samples) randomly. In training dataset, univariate Cox regression was used with the survival R package (p < 0.01) to identify survival-related IRGS, the Least Absolute Shrinkage and Selection Operator (LASSO) regression was used to eliminate collinearity among IRGs^29^ . Ultimately a prognostic model involving seven key IRGs was constructed by multivariate Cox proportional hatablezards regression analysis^30^. The immune risk score was served as predictors of prognostic status. And we calculated the immune risk score with the following formula: risk score  = $\sum\nolimits_{i = 1}^{n} {\exp i*\beta i.}$ .

We plotted the Kaplam-Meier survival curves to evaluate the model’s prediction effect. All of the samples were categorized into high-risk group and low-risk group according to the median value of the training dataset. Time-dependent receiver operating characteristic (ROC) curves were plotted to appraise the prediction performance of 1-, 3-, 5-year survival^31^ . We also calculated the Area Under roc curve (AUC) of the training dataset, testing dataset and GSE dataset via time ROC R package.

### Relationship between IRGs signature and clinicopathologic features

The univariate Cox analysis determined the correlation between survival and clinicopathologic features while the indicators including: age, gender, TNM stage, tumor grade and Immune risk score. Then the independent prognostic indicators of HCC was identified by the multivariate Cox analysis. Finally we generated a prognostic nomogram using rms R package.

### Relationship between IRGs signature and immune checkpoints expression

TIMER database (https://cistrome.shinyapps.io/timer/) is a public resource to analyze and visualize the abundance of tumor-infiltrating immune cells in a given cancer type^32^. In order to explore the effect of the IRGs signature in HCC immunotherapy, the “Correlation” module was used to calculate the relationship between the IRGs signature and another 6 immune checkpoints’ expression including PDCD1, PDCD1LG2, CTLA4, CD247, HAVCR2, and IDO1. And Spearman’s correlation > 0.3 was considered to have a significant correlation.

### Gene set enrichment analysis (GSEA)

The differences of signaling pathway were integrated through the Kyoto Encyclopedia of Genes and Genomes (KEGG) database^33^, and determined by gene set enrichment analysis (GSEA) using the software GSEA 4.0.3 (http://www.gsea-msigdb.org) with FDR < 0.01 and normalized enrichment score (NES) > 1.9.

## Results

### Identification of prognosis-related modules by WGCNA

**Figure 2:**
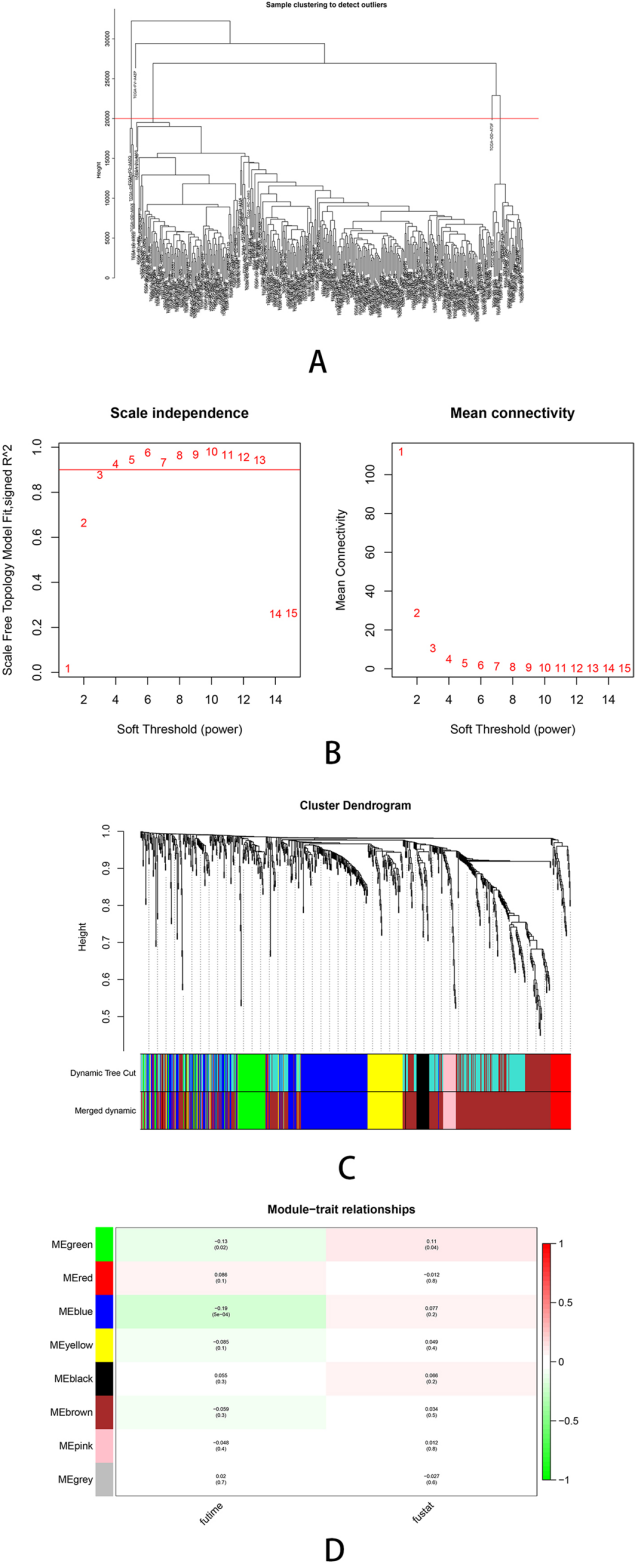
Weighted gene correlation network analysis. **(A)** The samples hierarchical clustering tree, height greater than 20,000 has been removed. **(B)** The scale‐free fit index for soft‐thresholding powers. The soft‐thresholding powers was identified based on a scale‐free R2 = 0.9. **(C)** The cluster dendrogram plotted by dynamic pruning method. Each branch in the figure represents one gene, each color below represents one co‐expression module. **(D)** The heatmap for the correlation between clinical traits and gene module.

Weighted Gene Correlation Network Analysis (WGCNA) was performed on 1284 overlapping immune-related genes (Fig. 1B). Firstly, we removed one sample, TCGA-FV-A4ZP, as the height in the hierarchical clustering tree is greater than 20,000 (Fig. 2A). The soft-thresholding power to construct a gene regulatory network was established basing on a scale-free R^2^ = 0.9 (Fig. 2B). Eight modules were identified using dynamic pruning method and similar modules has been merged (Fig. 2C). The highest correlation with survival status was shown in green module while the red and blue modules showed the highest correlation with the overall survival of HCC patients (Fig. 2D). Finally, we chose a total of 318 IRGs from the three modules for further analysis.

### Construction of prognostic signature based on immune related genes

**Figure 3:**
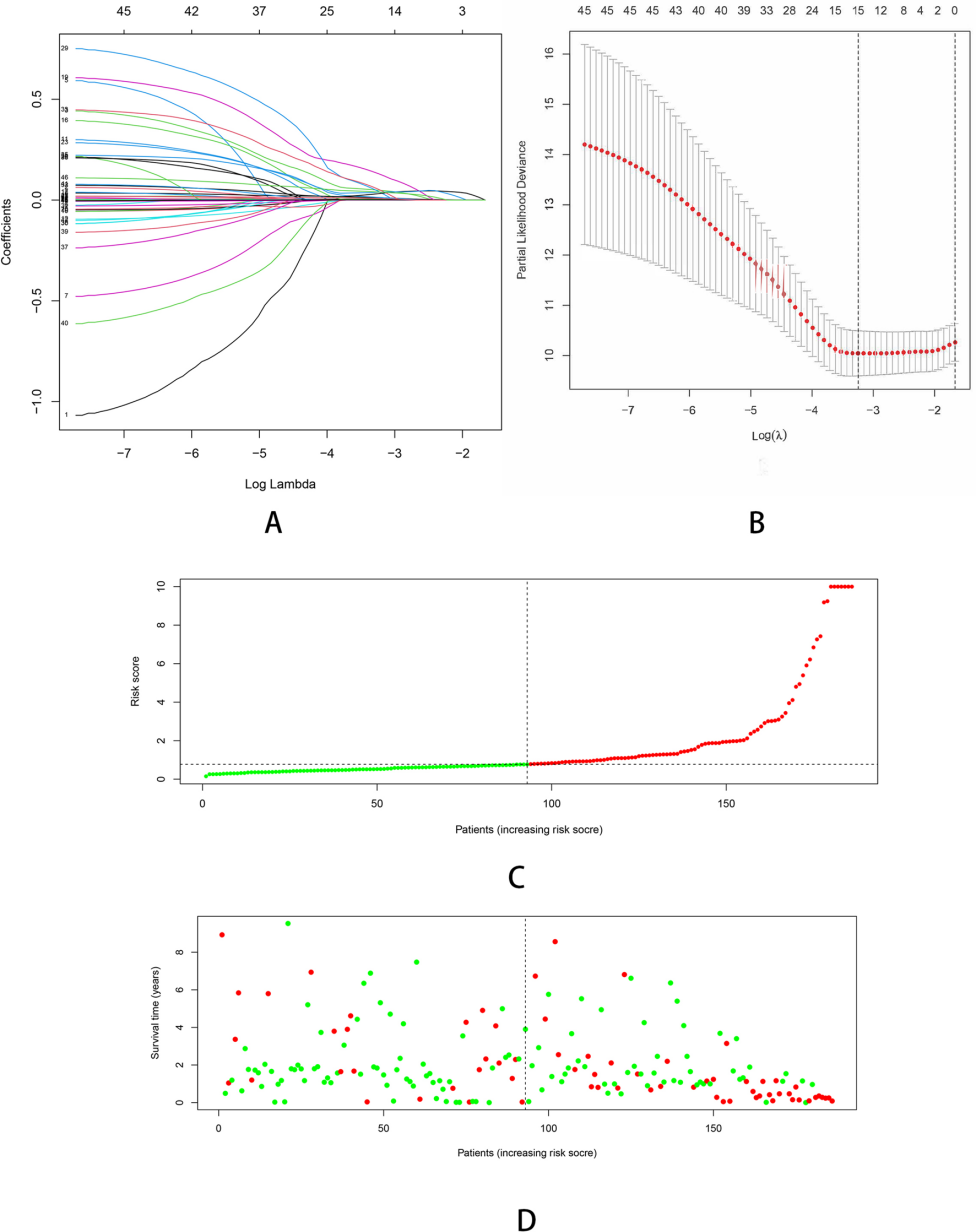
Construction of the immune-related prognostic signature in HCC. **(A)** Lasso regression analysis to eliminate collinearity. **(B)** Partial likelihood deviance for different numbers of variables. **(C)** The distribution of risk score. **(D)** The survival status and duration of patients.

**Figure 4:**
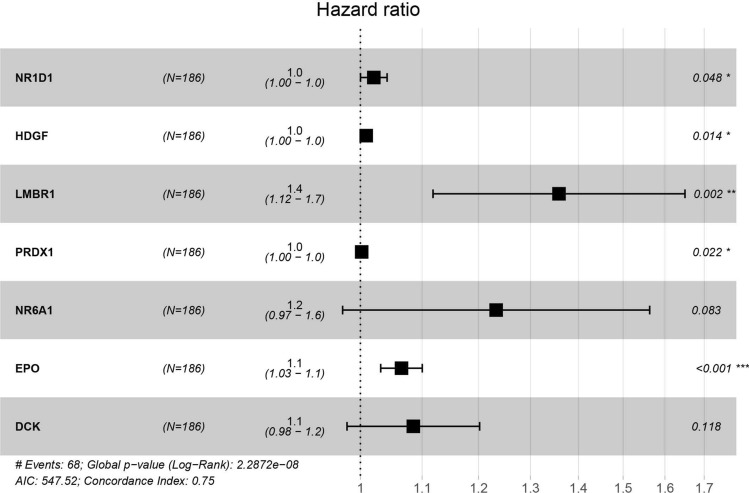
The IRGs in prognostic signature identified by multivariate Cox regression.

**Figure 5:**
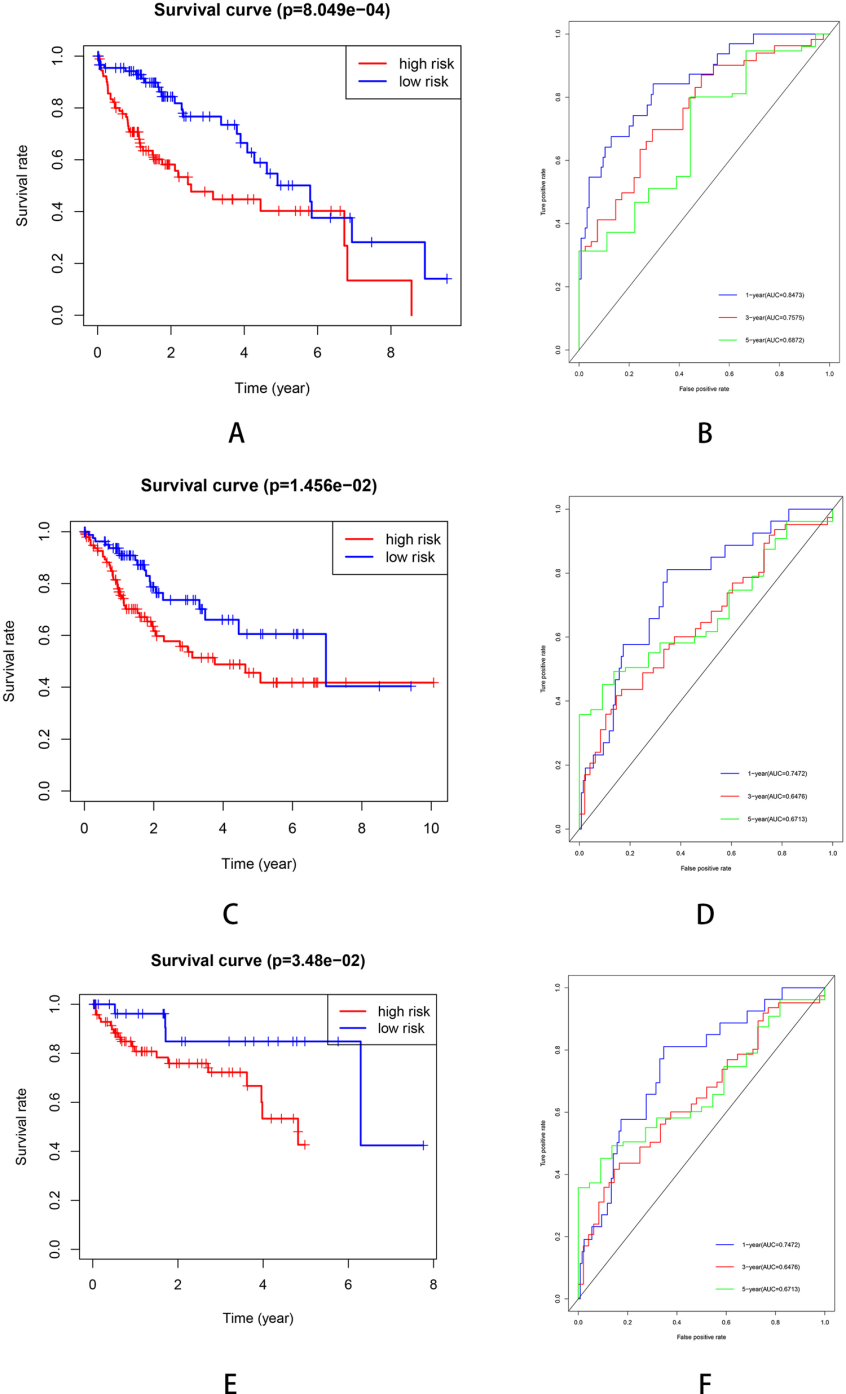
**(A)** Kaplan–Meier plots of TCGA training cohort. **(B)** ROC curve for the TCGA training cohort. **(C)** Kaplan–Meier plots curve of TCGA testing cohort. **(D)** ROC curve for the TCGA testing cohort. **(E)** Kaplan–Meier plots curve of GSE cohort. (F) ROC curve for the GSE cohort.

As the result of univariate Cox regression analysis, 46 survival related genes of the above three modules were identified with the cut-off value of P < 0.01 (Table s2). In order to eliminate collinearity between IRGs, Lasso regression analysis was performed (Fig. 3). In the end, a total of 7 genes (NR1D1, HDGF, LMBR1, PRDX1, NR6A1, EPO and DCK) were included in multivariate Cox regression to establish a prognostic signature. The hazard ratio and 95%CI of the seven IRGs are presented (Fig. 4). The Kaplan–Meier curves were plotted in the training dataset according to the immune risk score and the high-risk group showed a poor overall survival compared to low-risk group (Fig. 5A). The time-dependent ROC curves revealed the prognostic signature had a superior predictive accuracy as the AUC was 0.8473 for 1-year, 0.7575 for 3-year, 0.6872 for 5-year in the training dataset (Fig. 5B).

### Verification of the prognostic signature in testing dataset and GSE cohort

**Figure 6:**
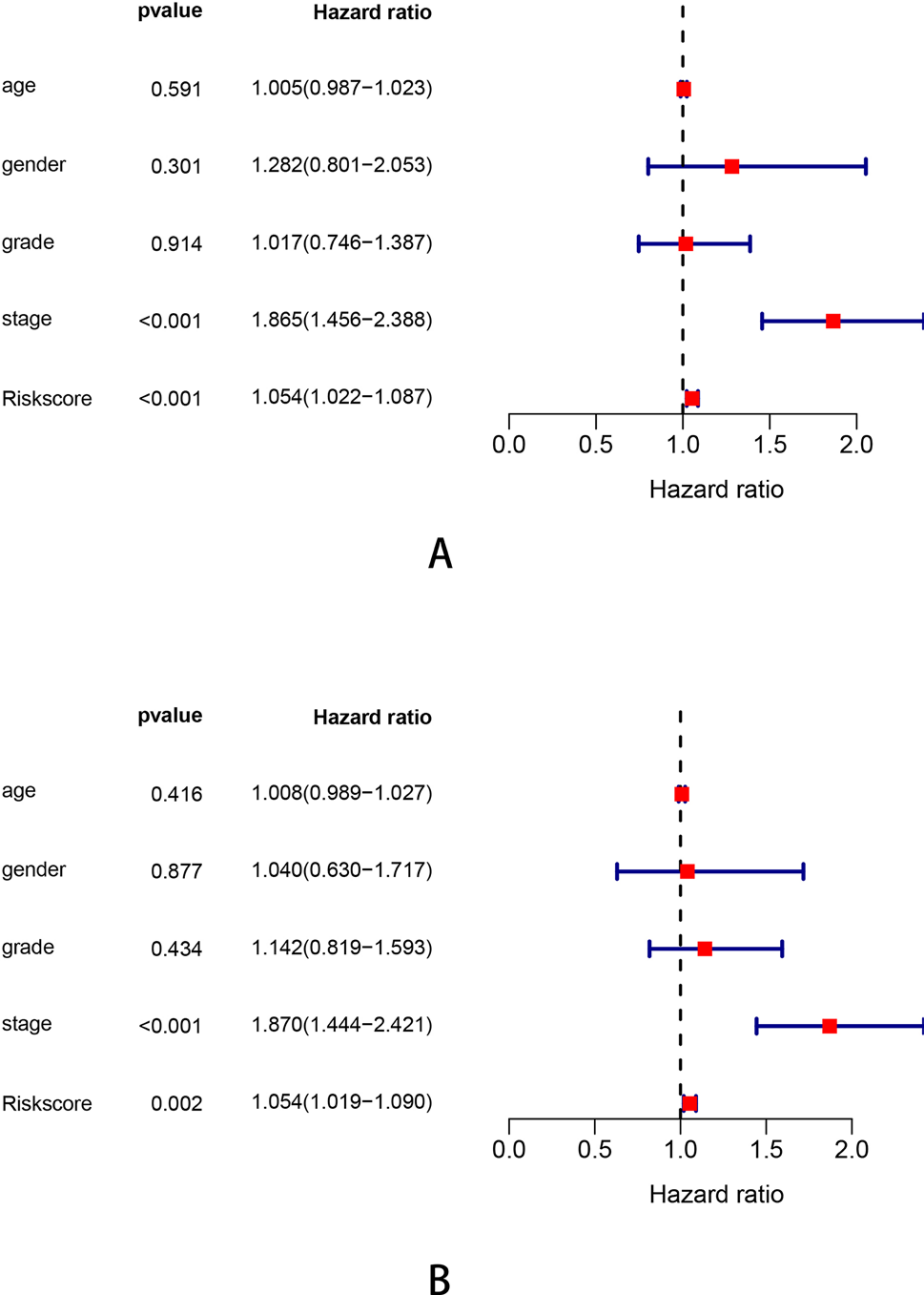
The univariate **(A)** and multivariate **(B)** Cox proportional hazards regression for Immune risk score and clinical factors.

**Figure 7:**
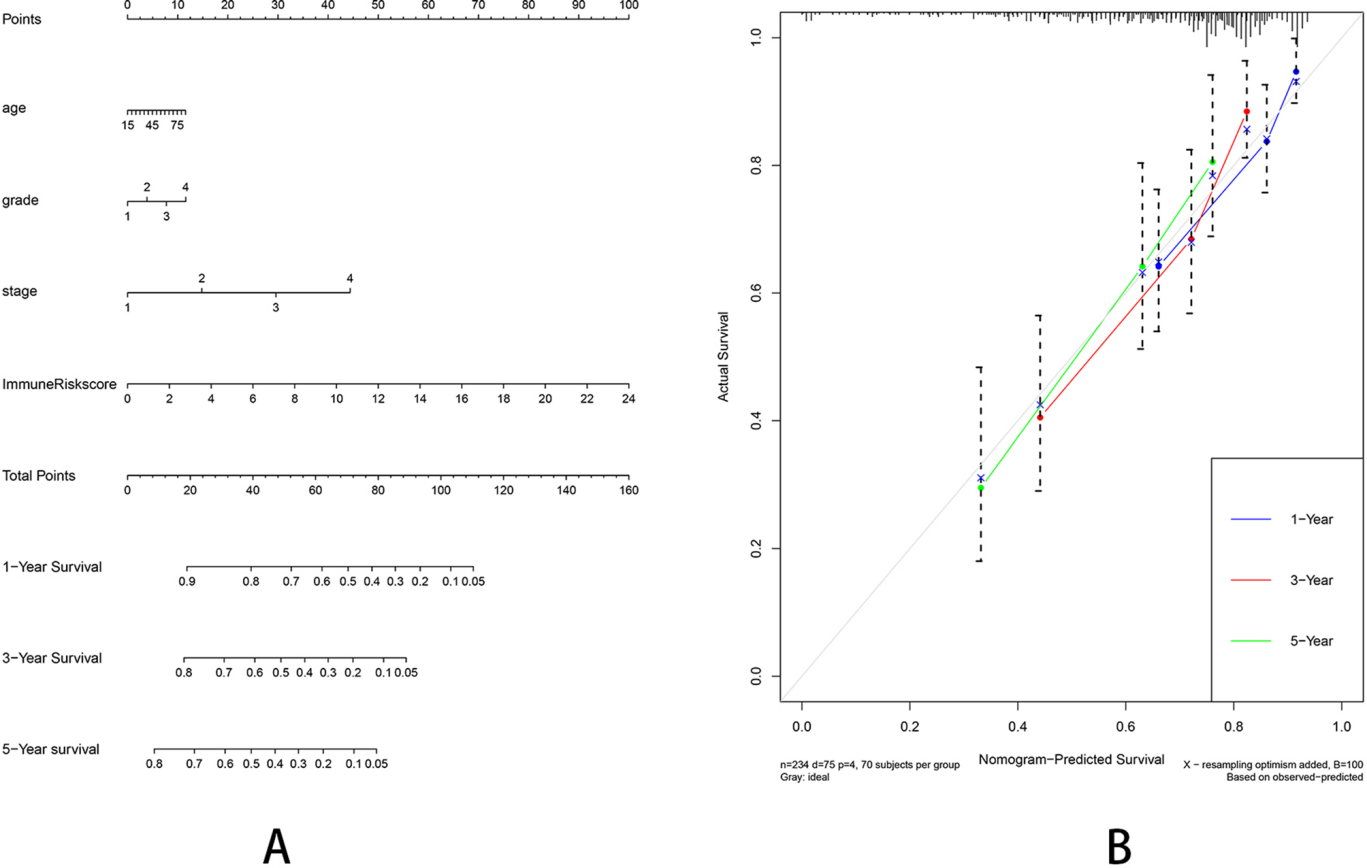
**(A)** A prognostic nomogram including Immune-Risk score and other clinical factors. **(B)** The calibration plot comparing predicted and actual 1-, 3-, 5-year overall survival. The graph relative to the 45 diagonal reveals the model relative to perfect prediction.

The clinical characteristics of patients from TCGA and GEO database are shown in Table 1. In the TCGA testing dataset, the Kaplan–Meier analysis revealed that the high-risk patients showed a worse survival (Fig. 5C). The time-dependent ROC curves are plotted (Fig. 5D). We also used an independent cohort (GSE76427) to verify the predictive ability of the seven-gene signature. The Kaplan–Meier curve in GSE cohort was plotted (Fig. 5E) and the ROC curve showed the signature still had a good accuracy as the AUC was 0.6887 for 1-year, 0.604 for 3-year and 0.6332 for 5-year (Fig. 5F). Moreover, the immune-risk signature was an independent prognostic indicator for overall survival (OS) in multivariate Cox analysis with p < 0.01 (Fig. 6). We also constructed a prognostic nomogram which integrating the clinical features with immune-risk score as a quantitative tool for predicting OS of HCC patients (C index = 0.714, 95% CI = 0.651–0.777, Fig. 7A). The calibration plots presented that the nomogram performed a moderate accuracy (Fig. 7B).

**Table 1:** The clinical characteristics of patients from TCGA and GEO database.

Variable	Train (n = 176)	Test (n = 169)	GSE (n = 114)	P value
Median age (IQR)	61 (20–84)	59 (16–85)	64 (14–93)	0.0020
**Gender**				
Male	116 (65.9%)	118 (69.8%)	93 (80.9%)	0.0039
Female	60 (34.1%)	51 (30.2%)	21 (19.1%)	0.0251
**Grade**				
Gl–G2	112 (63.6%)	103 (60.9%)	–	0.0138
G3–G4	64 (36.4%)	66(39.1%)	–	0.0228
**BCLC stage**				
A	*–*	*–*	77 (67.5%)	
B–C	*–*	*–*	37 (32.5%)	
**TNM stage**				
T1–T2	134 (76.1%)	121 (71.6%)	90 (78.9%)	0.0008
T3–T4	42 (23.9%)	48 (28.4%)	24 (21.1%)	0.0075
Median overall survival (years)	1.3 (0–9.5)	1.5 (0–10.1)	1.2(0–7.8)	0.3610
**Events**				
Live	118 (67.0%)	115 (68.0%)	91 (79.8%)	0.0033
Death	58 (33.0%)	54 (32.0%)	23 (20.2%)	0.0203

### The IRGs signature’s effect in HCC immunotherapy

**Figure 8:**
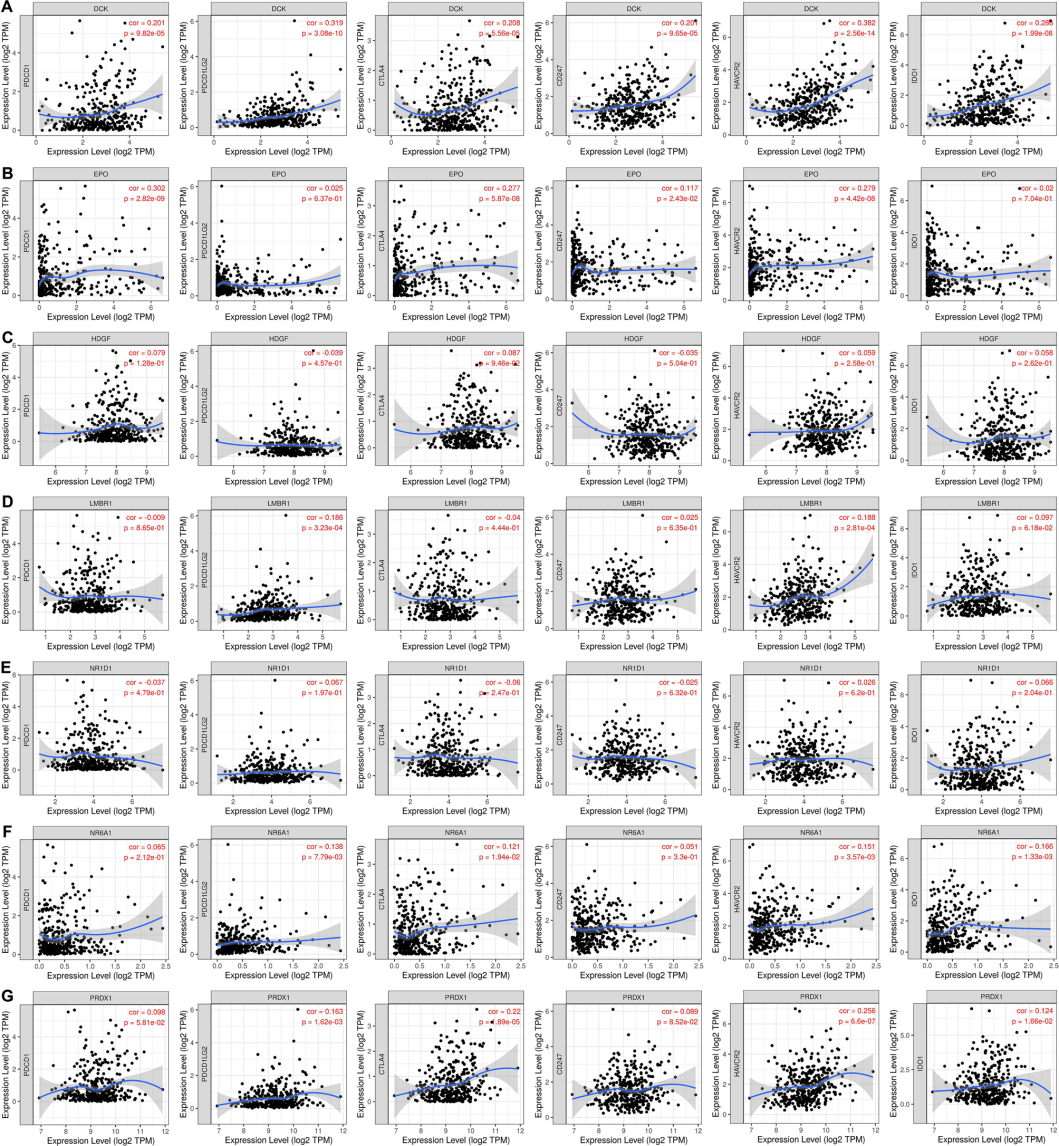
The expression scatterplots of IRGs and another 6 immune checkpoints based on TIMER database analysis.

We used the TIMER database to further explore the relationship between the IRGs signature and the expression of another 6 immune checkpoints including PDCD1, PDCD1LG2, CTLA4, CD247, HAVCR2, and IDO1. The expression scatterplots were plotted (Fig. 8). According to the results, the expression of DCK was significantly associated with PDCD1LG2 (cor = 0.319, P < 0.05) and HACVR2 (cor = 0.382, P < 0.05). And the expression of EPO was significantly associated with PDCD1 (Spearman’s correlation = 0.302, P < 0.05), which indicates that DCK and EPO might play a important role in the response to immunotherapy in HCC.

### Gene set enrichment analysis (GSEA)

**Figure 9:**
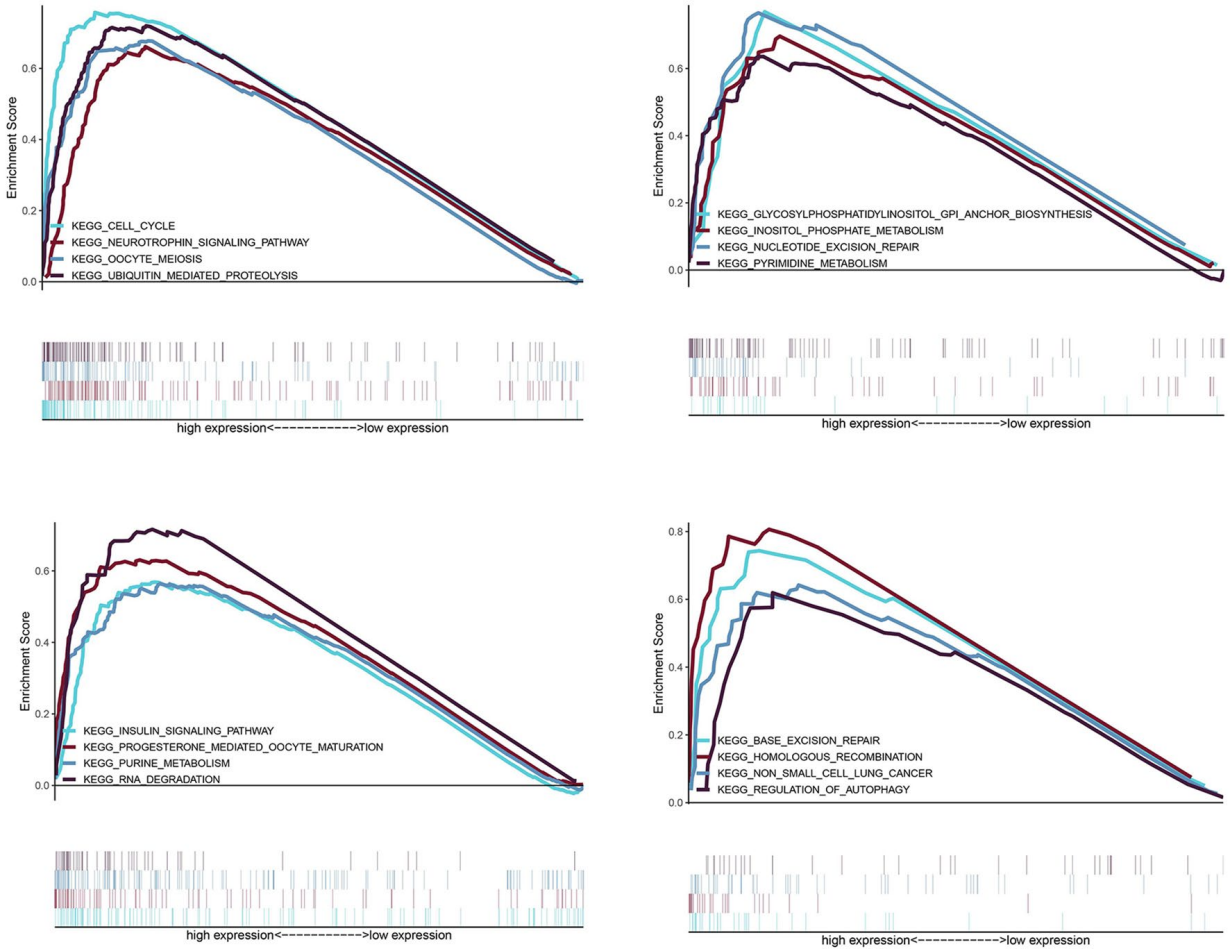
GSEA analysis, 16 KEGG pathways were significantly activated in high-risk group.

GSEA analysis identified 16 significant KEGG pathways including: oocyte meiosis, cell cycle, ubiquitin mediated proteolysis, neurotrophin signaling pathway, glycosylphosphatidylinositol (GPI) anchor biosynthesis, pyrimidine metabolism, inositol phosphate metabolism, nucleotide excision repair, insulin signaling pathway, RNA degradation, purine metabolism, progesterone mediated oocyte maturation, regulation of autophagy, excision repair and non-small cell lung cancer. All of these pathways were differentially activated between high- and low-risk groups (Fig. 9).

## Discussion

HCC is one of the most deadly cancers all around the world, whose incidence and mortality rates are predicted to continue rising^34^. Liver resection, liver transplantation and radiofrequency ablation have been the potential curative treatments for HCC patients at a relatively early stage for a long time^35–38^. Transarterial chemoembolization and Tyrosine kinase inhibitor targeting agent Sorafenib have been demonstrated having survival benefits advanced HCC patients. While the improvement of overall survival is still limited. Sorafenib can only prolong the overall survival of advanced HCC patients by 3 months, and it also has significant side reaction.

Immunotherapy is developing rapidly and has formed part of the standard treatment regimen for many cancer diseases^39^. The normal liver is a tolerogenic immune organ in order to prevent the abnormal immune response caused by continual pathogen exposure^40^. Chronic inflammatory, which can inhibit the antigen-specific immune surveillance, is an important process in the development of HCC^41^. Chronic inflammatory state also leads to the overexpression of immune checkpoint molecules^42^. Therefore, activate the immune reaction to HCC can be helpful to the treatment. Several studies showed that increasing the level of activated peripheral blood mononuclear cells and dendritic cells benefited in both early and advanced stage HCC^43–45^. The blockade of immune checkpoints is the most successful immunotherapeutic in various cancer diseases. Programmed cell death-1(PD-1) antibodies including nivolumab and pembrolizumab have already shown promising efficacy in advanced HCC patients^46^. However, the reliable predictive biomarkers for immunotherapy are still lacking.

High throughput sequencing technology, gene expression databases and bioinformatics analysis provided possibility for systematic profiling of immune signatures in solid cancer. Wang et al.^22^ constructed an immune-related prognostic signature including FGF2, SCL10A2, NDRG1, CCL28, UCN, ESM1, UTS2 and TRDC for colorectal cancer. Another bioinformatics research which analyzed three circRNA datasets and one miRNA dataset found that UBE2L3 was the key gene in the pathogenesis of HCC^47^. In this study, we identified a seven-gene signature comprising NR1D1, HDGF, LMBR1, PRDX1, NR6A1, EPO and DCK that was closely related to the prognosis. NR6A1 and NR1D1 were members of the nuclear hormone receptor family^48^. NR6A1 was reported as a novel biomarker associated with migration and invasion of prostate cancer^49^. The overexpression of NR6A1 in prostate cancer cells could reduce G0/G1 phase cell cycle arrest and promoted tumor growth^50^. Hepatoma-derived growth factor (HDGF) played an important role in the development and progression in many solid cancers including HCC^51,52^. HDGF stimulated the differentiation of neutrophils in gastric cancer patients and relayed Hp-induced inflammatory signaling, which was involved in gastric carcinogenesis^53^. High HDGF levels in serum of non-small cell lung cancer patients also indicated bone metastasis and unfavorable prognosis^54^. LMBR1 was related to the preaxial polydactyly in human and mouse. Its role in the tumor progression is still unclear. While according to a gene expression study about gastrointestinal stromal tumor, LMBR1 might be a tumor progression promoter in GISTs by regulating the expression of nuclear BMI1^55^. PRDX1 was a member of peroxiredoxin family^56^, whose overexpression was associated with lymph metastasis, histopathological grading and the tumor size in head and neck squamous cell carcinoma patients^57^. Bajor et al.^58^ also found that the downregulation of PRDX1 could significantly impaired the growth rate of breast cancer cells and was a potential target for breast cancer treatment. Erythropoietin (EPO) was a biomarker of predicting prognosis in patients with Acute-on-chronic liver failure (ACLF), and patients without bleeding showed a lower level of EPO^59^. Deoxycytidine kinase (DCK) was well-known for its association with resistance to chemotherapeutic agents. Based on the research of Shang et al.^60^, the knock down of DCK inhibited proliferation and tumorigenicity of cervical cancer cells.

PDCD1 encodes the Programmed cell death protein-1 (PD-1), which expresses in the activated T cells. The interaction between PD-1 and its ligand PD-L1 (encoded by PDCD1LG1) suppresses the activation of lymphocytes and cytokine production^61^. Inhibitors of PD-1 and PD-L1 have shown great clinical benefits in some types of cancer^62^. Our study analyzed the correlation between the expression of IRGs and immune check point and found that the expression of PDCD1/PDCD1LG2 was in positive correlation with the expression of DCK and EPO, which revealed that the overexpression of DCK and EPO may upregulate the expression of PD-1/PD-L1, and lead to a suppression of anti-tumor immune response. The expression of DCK was also in positive correlation with the expression of HAVCR2. HAVCR2, which also known as TIM3, plays an important role in inhibiting the Th1 response and the expression of cytokines, some preclinical researches have shown that vivo blockade of Tim-3 leading to the enhance of anti-tumor immunity and the inhibition of tumor growth^63^. The expression level of DCK was positively related to TIM3, indicating a potential correlation with the immunological tolerance in HCC. Thus, the DCK and EPO from IRGs may regulate the expression of some specific immune checkpoints and promote the tumor escape mechanisms in HCC. Further researches are needed to explore whether DCK and EPO could be the potential targets in immune checkpoint inhibitor therapy.

Gene set enrichment analysis (GSEA) indicated 16 significant differential KEGG pathways among high-and low-risk groups. Cell cycle is accomplished through a series of events including S phase, M phase, G1 and G2 phases^64^. The check point network controlled by ATM/ATR and CHK2/CHK1 can prolong cell-cycle progression in G1, G2 or S phases in respond to DNA damage^65–68^. However, mutations in checkpoint pathway provide the opportunity for the continued growth of genomic abnormalities cells, thereby increasing the chance of malignant transformation^69^. Glycosylphosphatidylinositol (GPI)-anchored proteins has been reported associated with a range of cancer^70^. Trink et al. found the first oncogenic GPI-T subunit, PIG-U, in bladder cancer. Another research showed that the level of cell surface GPI-anchored protein is high in breast cancer cells^71^. Nucleotide Excision Repair pathway plays a crucial role in cancer prevention^72^. And the defects of the global genome NER (GG-NER) sub pathway result in cancer predisposition^73^. Autophagy plays an important role in controlling protein and organelle quality and quantity^74^,which can promote cancer progression by suppressing P53 expression and inhibiting cell death, senescence, and an anti-tumor immune response. In addition, the homologous recombination repair (HRR) pathway provides high-fidelity repair of double-strand DNA breaks. The deficiency in HRR is a target for a new selective therapy for high-grade ovarian cancer^75^.

Our study has following limitations. Firstly, the number of samples we retrieved from TCGA database and GSE76427 are relatively small, so that the prediction ability of our IRGs prognostic signature should be verified in a large prospective clinical cohort. Secondly, as the clinical information released in publicly available datasets is limited, the clinicopathological parameters that included in nomogram were not comprehensive. The overall survival of HCC patients are significantly impacted by some other clinical factors as well. Including the aetiology of HCC (HBV/HCV infection), the status of liver failure and portal hypertension, the treatment received by HCC patients and so on. Based on this limitation, the immune-related signatures may not be an absolute independent risk factor for the overall survival for HCC patients. Variables not included in our study may have potential relevance to the immune-related signatures, which need to be further explored in specific research. Thirdly, our study was a retrospective analysis based on the data from published studies rather than real-word treatment experience, therefore the clinical application value of the IRGs prognostic signature needed further evaluation. What’s more, the IRGs prognosis signature in this study was established by pure bioinformatics analysis. Therefore, further experiments are needed to validate our results.

In conclusion, we successfully recognized a novel IRGs prognostic signature with high predictive value and accuracy for patients with HCC, which may contribute to the decision making of clinical management. Moreover, this study provides additional information for further research on HCC pathogenesis and targeted therapies.

## Supplementary Information

Supplementary Tables.

## Data Availability

The datasets supporting the conclusions of this article are available in the public databases TCGA database (http://www.cancer.gov/tcga) and Gene Expression Omnibus (https://www.ncbi.nlm.nih.gov/gds/) with the accession numbers: GSE76427.

## References

[1] Couri T, Pillai A (2019). Goals and targets for personalized therapy for HCC. Hepatol. Int..

[2] Chaudhary K (2018). Deep learning-based multi-omics integration robustly predicts survival in liver cancer. Clin. Cancer Res..

[3] Forner A, Llovet JM, Bruix J (2012). Hepatocellular carcinoma. Lancet.

[4] Xie Y (2018). Immunotherapy for hepatocellular carcinoma: Current advances and future expectations. J. Immunol. Res..

[5] Greten TF (2019). Targeted and immune-based therapies for hepatocellular carcinoma. Gastroenterology.

[6] Llovet JM (2008). Sorafenib in advanced hepatocellular carcinoma. N. Engl. J. Med..

[7] Lencioni R (2014). GIDEON (Global Investigation of therapeutic DEcisions in hepatocellular carcinoma and Of its treatment with sorafeNib): Second interim analysis. Int. J. Clin. Pract..

[8] Forner A, Reig M, Bruix J (2018). Hepatocellular carcinoma. Lancet.

[9] Waidmann O (2018). Recent developments with immunotherapy for hepatocellular carcinoma. Expert Opin. Biol. Ther..

[10] El-Khoueiry AB (2017). Nivolumab in patients with advanced hepatocellular carcinoma (CheckMate 040): An open-label, non-comparative, phase 1/2 dose escalation and expansion trial. Lancet.

[11] Gordan JD (2020). Systemic therapy for advanced hepatocellular carcinoma: ASCO guideline. J. Clin. Oncol..

[12] Finn RS (2020). Atezolizumab plus bevacizumab in unresectable hepatocellular carcinoma. N. Engl. J. Med..

[13] Xu W (2019). Immunotherapy for hepatocellular carcinoma: Recent advances and future perspectives. Ther. Adv. Med. Oncol..

[14] Ribas A (2013). Hepatotoxicity with combination of vemurafenib and ipilimumab. N. Engl. J. Med..

[15] Amin A (2019). Correction to: Safety and efficacy of nivolumab in combination with sunitinib or pazopanib in advanced or metastatic renal cell carcinoma: the CheckMate 016 study. J. Immunother. Cancer.

[16] Yarchoan M (2019). Recent developments and therapeutic strategies against hepatocellular carcinoma. Cancer Res..

[17] Deng J (2019). Identification of the germline mutation profile in esophageal squamous cell carcinoma by whole exome sequencing. Front. Genet..

[18] Dong, X. *et al*. Upregulation of LAGE3 correlates with prognosis and immune infiltrates in colorectal cancer: A bioinformatic analysis. *Int. Immunopharmacol.* **85**, 106599 (2020). 10.1016/j.intimp.2020.106599 32438075

[19] Gong SQ (2019). The expression and effection of microRNA-499a in high-tobacco exposed head and neck squamous cell carcinoma: A bioinformatic analysis. Front. Oncol..

[20] Liu J (2019). Identification of EPHX2 and RMI2 as two novel key genes in cervical squamous cell carcinoma by an integrated bioinformatic analysis. J. Cell Physiol..

[21] Xia L (2019). Integrated bioinformatic analysis of a competing endogenous RNA network reveals a prognostic signature in endometrial cancer. Front. Oncol..

[22] Huang R (2020). A novel immune-related genes prognosis biomarker for melanoma: associated with tumor microenvironment. Aging (Albany NY).

[23] Wang J (2020). A novel prognostic signature of immune-related genes for patients with colorectal cancer. J. Cell Mol. Med..

[24] Song Q (2019). Identification of an immune signature predicting prognosis risk of patients in lung adenocarcinoma. J. Transl. Med..

[25] Yang W (2019). Immune signature profiling identified prognostic factors for gastric cancer. Chin. J. Cancer Res..

[26] Song K, Li L, Zhang G (2017). Bias and correction in RNA-seq data for marine species. Mar. Biotechnol. (NY).

[27] Bhattacharya S (2014). ImmPort: Disseminating data to the public for the future of immunology. Immunol. Res..

[28] Zhang, B. & Horvath, S. A general framework for weighted gene co-expression network analysis. *Stat. Appl. Genet. Mol. Biol*. **4**, 17 (2005). 10.2202/1544-6115.1128 16646834

[29] Shahraki HR, Salehi A, Zare N (2015). Survival prognostic factors of male breast cancer in southern Iran: A LASSO-Cox regression approach. Asian Pac. J. Cancer Prevent..

[30] Liang H (2004). Multivariate Cox analysis on prognostic factors after surgery for rectal carcinoma. Zhonghua Zhong Liu Za Zhi.

[31] Schemper M, Henderson R (2000). Predictive accuracy and explained variation in Cox regression. Biometrics.

[32] Li T (2017). TIMER: A web server for comprehensive analysis of tumor-infiltrating immune cells. Cancer Res..

[33] Kanehisa M (2021). KEGG: Integrating viruses and cellular organisms. Nucleic Acids Res..

[34] Jemal, A. *et al*. Annual report to the nation on the status of cancer, 1975–2014, featuring survival. *J. Natl. Cancer Inst.* **109**(9) (2017). 10.1093/jnci/djx030 PMC5409140 28376154

[35] Yarchoan M (2019). Correction: Recent developments and therapeutic strategies against hepatocellular carcinoma. Cancer Res..

[36] Facciorusso A (2015). Transarterial chemoembolization: Evidences from the literature and applications in hepatocellular carcinoma patients. World J. Hepatol..

[37] Habib A (2015). Locoregional therapy of hepatocellular carcinoma. Clin. Liver Dis..

[38] Shin JW, Chung YH (2013). Molecular targeted therapy for hepatocellular carcinoma: Current and future. World J. Gastroenterol..

[39] Pagni, F. *et al*. Targeting immune-related biological processes in solid tumors: We do need biomarkers. *Int. J. Mol. Sci.* **20**(21) (2019). 10.3390/ijms20215452 PMC6862285 31683784

[40] Pardee AD, Butterfield LH (2012). Immunotherapy of hepatocellular carcinoma: Unique challenges and clinical opportunities. Oncoimmunology.

[41] Makarova-Rusher OV (2015). The yin and yang of evasion and immune activation in HCC. J. Hepatol..

[42] Hato T (2014). Immune checkpoint blockade in hepatocellular carcinoma: Current progress and future directions. Hepatology.

[43] Kayashima H (2010). Intratumoral neoadjuvant immunotherapy using IL-12 and dendritic cells is an effective strategy to control recurrence of murine hepatocellular carcinoma in immunosuppressed mice. J. Immunol..

[44] Lee WC (2005). Vaccination of advanced hepatocellular carcinoma patients with tumor lysate-pulsed dendritic cells: A clinical trial. J. Immunother..

[45] Takayama T (2000). Adoptive immunotherapy to lower postsurgical recurrence rates of hepatocellular carcinoma: A randomised trial. Lancet.

[46] Kim DW, Talati C, Kim R (2017). Hepatocellular carcinoma (HCC): Beyond sorafenib-chemotherapy. J. Gastrointest. Oncol..

[47] Wu J (2019). Bioinformatic analysis of circular RNA-associated ceRNA network associated with hepatocellular carcinoma. Biomed. Res. Int..

[48] Garattini E (2016). Lipid-sensors, enigmatic-orphan and orphan nuclear receptors as therapeutic targets in breast-cancer. Oncotarget.

[49] Mathieu R (2013). Expression screening of cancer/testis genes in prostate cancer identifies NR6A1 as a novel marker of disease progression and aggressiveness. Prostate.

[50] Cheng G (2017). Positive expression of NR6A1/CT150 as a predictor of biochemical recurrence-free survival in prostate cancer patients. Oncotarget.

[51] Yang, F. *et al.* Downregulated expression of hepatoma-derived growth factor inhibits migration and invasion of prostate cancer cells by suppressing epithelial-mesenchymal transition and MMP2, MMP9. *PLoS One* **13**(1), e0190725 (2018). 10.1371/journal.pone.0190725 PMC5754131 29300772

[52] Wang Y (2020). Development and validation of a prognostic and immunotherapeutically relevant model in hepatocellular carcinoma. Ann. Transl. Med..

[53] Chu TH (2019). Hepatoma-derived growth factor participates in *Helicobacter pylori*-induced neutrophils recruitment, gastritis and gastric carcinogenesis. Oncogene.

[54] Zhang G (2017). High serum HDGF levels are predictive of bone metastasis and unfavorable prognosis in non-small cell lung cancer. Tohoku J. Exp. Med..

[55] Bai C (2018). Expression profiles of stemness genes in gastrointestinal stromal tumor. Hum. Pathol..

[56] Kim EK (2019). Peroxiredoxin 1 post-transcriptionally regulates snoRNA expression. Free Radic. Biol. Med..

[57] Jiang Y (2019). LncRNA LINC00460 promotes EMT in head and neck squamous cell carcinoma by facilitating peroxiredoxin-1 into the nucleus. J. Exp. Clin. Cancer Res..

[58] Bajor M (2018). Targeting peroxiredoxin 1 impairs growth of breast cancer cells and potently sensitises these cells to prooxidant agents. Br. J. Cancer.

[59] Alempijevic T (2016). Erythropoietin in predicting prognosis in patients with acute-on-chronic liver failure. J. Gastrointest. Liver Dis..

[60] Shang QY, Wu CS, Gao HR (2017). Effects of DCK knockdown on proliferation, apoptosis and tumorigenicity in vivo of cervical cancer HeLa cells. Cancer Gene Ther..

[61] Kasamatsu T (2020). PDCD1 and PDCD1LG1 polymorphisms affect the susceptibility to multiple myeloma. Clin. Exp. Med..

[62] Yarchoan, M. *et al*. PD-L1 expression and tumor mutational burden are independent biomarkers in most cancers. *JCI Insight* **4**(6) (2019). 10.1172/jci.insight.126908 PMC6482991 30895946

[63] Das M, Zhu C, Kuchroo VK (2017). Tim-3 and its role in regulating anti-tumor immunity. Immunol. Rev..

[64] Tessema M, Lehmann U, Kreipe H (2004). Cell cycle and no end. Virchows Arch..

[65] Bartek J, Lukas J (2003). Chk1 and Chk2 kinases in checkpoint control and cancer. Cancer Cell.

[66] Bartek J, Lukas C, Lukas J (2004). Checking on DNA damage in S phase. Nat. Rev. Mol. Cell Biol..

[67] Nyberg KA (2002). Toward maintaining the genome: DNA damage and replication checkpoints. Annu. Rev. Genet..

[68] Xu B (2002). Two molecularly distinct G(2)/M checkpoints are induced by ionizing irradiation. Mol. Cell Biol..

[69] Kastan MB, Bartek J (2004). Cell-cycle checkpoints and cancer. Nature.

[70] Gamage DG, Hendrickson TL (2013). GPI transamidase and GPI anchored proteins: Oncogenes and biomarkers for cancer. Crit. Rev. Biochem. Mol. Biol..

[71] Guo Z (2004). CDC91L1 (PIG-U) is a newly discovered oncogene in human bladder cancer. Nat. Med..

[72] Liakos A, Lavigne MD, Fousteri M (2017). Nucleotide excision repair: From neurodegeneration to cancer. Adv. Exp. Med. Biol..

[73] Marteijn JA (2014). Understanding nucleotide excision repair and its roles in cancer and ageing. Nat. Rev. Mol. Cell Biol..

[74] Maheswari U, Sadras SR (2018). Mechanism and regulation of autophagy in cancer. Crit. Rev. Oncogene.

[75] Ledermann JA, Drew Y, Kristeleit RS (2016). Homologous recombination deficiency and ovarian cancer. Eur. J. Cancer.

